# Effects of a Commercial Fruit, Vegetable and Berry Juice Powder Concentrate on Metabolic Safety and Cardiovascular Health in Older Adults: 6-Month Results of a Randomized Controlled Trial

**DOI:** 10.3390/nu18172746

**Published:** 2026-08-22

**Authors:** Tobias Ziegler, Azra Darko, Gerhard Ledinski, Gerd Kager, Gerd Hörl, Andreas Seemann, Gerhard Cvirn, Manfred Lamprecht

**Affiliations:** 1 Division of Medicinal Chemistry, Otto Loewi Research Center, Medical University of Graz, 8010 Graz, Austria; tobias.ziegler@greenbeat.at (T.Z.); azra.darko@medunigraz.at (A.D.); gerhard.ledinski@medunigraz.at (G.L.); gerd.kager@medunigraz.at (G.K.); gerd.hoerl@medunigraz.at (G.H.); manfred.lamprecht@greenbeat.at (M.L.); 2 Green Beat Institute of Nutrient Research, 8042 Graz, Austria; 3 Department of Human Movement Sciences, Sport and Health, University of Graz, 8010 Graz, Austria; andreas.seemann@greenbeat.at

**Keywords:** fruit and vegetable supplement, oxidative stress, homocysteine, older adults, randomized controlled trial, cardiovascular risk, safety

## Abstract

**Background/Objectives**: Fruit, vegetable and berry (FVB) juice powder concentrates are increasingly used to support cardiovascular health. Since they are usually consumed over long periods, this study assessed the metabolic safety of a commercial FVB supplement and its impact on selected biomarkers of systemic oxidative stress and cardiovascular health. **Methods**: In this open-label, randomized controlled trial, generally healthy older adults were allocated to a control group (*n* = 16) or an FVB group (*n* = 18), ingesting six capsules/day. This work reports the pre-specified 6-month timepoint of a 1-year trial. The primary outcome, metabolic safety, was assessed by hepatic, renal, thyroid, lipid and glucose metabolism markers. Exploratory analysis assessed carbonyl proteins, malondialdehyde, and oxidized LDL as markers of systemic oxidative stress, together with homocysteine and blood pressure as markers of cardiovascular health, while plasma vitamin C served as a compliance marker. **Results**: The FVB concentrate was well tolerated, and no safety-relevant deteriorations were observed in the FVB group. Carbonyl proteins decreased significantly in the FVB group (interaction *p* = 0.030). Homocysteine remained stable in the FVB group but increased significantly in the control group (interaction *p* = 0.002). Furthermore, systolic blood pressure decreased significantly (interaction *p* = 0.036), with a comparable interaction of diastolic blood pressure (*p* = 0.011), and plasma vitamin C increased significantly in the FVB group (interaction *p* = 0.004). After adjustment for baseline values, age and BMI, between-group differences persisted for homocysteine and diastolic blood pressure, whereas the differences in systolic blood pressure and carbonyl proteins were attenuated and no longer reached statistical significance. **Conclusions**: Six months of supplementation with a commercial FVB concentrate was metabolically safe and well tolerated. Since these findings are based on a pre-specified timepoint and a limited number of participants, they are hypothesis-generating rather than confirmatory and require confirmation by the complete trial. Registered at ClinicalTrials.gov (NCT04763291; registered on 21 February 2021).

## 1. Introduction

The safety of dietary supplements often does not get the attention it should, and concerns arise from impurities and contamination. The market is flooded with thousands of supplements, but not all of them are responsibly tested for quality and safety. In particular, plant-based formulations or extracts may be prone to residues from environmental exposure, including pesticide residues acquired during cultivation or harvesting, as well as remaining solvents originating from extraction or manufacturing processes. In addition, chemical contaminants may be introduced during post-harvest handling, storage, or processing. Identification of such contaminants depends on applied quality control measures, which means that these are not always identified or determined [1,2,3]. It is evident that environmental exposures such as pesticide residues have been associated with alterations in metabolic and cardiovascular biomarkers, even in generally healthy populations [4]. Beyond short-term issues related to product purity and contamination, the safety of plant-based dietary supplements also involves potential long-term metabolic effects of such compounds.

Excessive long-term consumption of naturally occurring bioactives present in plant-based extracts, often regarded as health-beneficial for a broad population, may even have a negative health impact and may therefore warrant investigation of hepatic and renal safety [5,6]. Changes in parameters of hepatic health have been reported to be affected by chronic exposure to certain chemical residues as well as bioactives/phytonutrients, indicating safety assessments should capture longer periods of time rather than acute toxicity evaluation [6,7]. For example, high-dose green tea extract supplementation over 12 months has been associated with increases in liver enzyme levels in a subset of otherwise healthy individuals [6].

Only a few clinical studies included assessment of hepatic, renal, thyroid, and cardiovascular health-related biomarkers, as well as biomarkers of oxidative stress after long-term consumption of plant-based extracts [8,9,10]. Oxidative stress increases with age and contributes to the pathogenesis of cardiovascular disease [11] and hence, oxidative stress markers are of particular importance for populations such as older adults or athletes [9,11,12]. In addition, elevated homocysteine levels represent another well-established risk factor for cardiovascular disease and are commonly observed in older adults [13].

Several clinical trials have already investigated FVB juice powder concentrates and reported favorable effects on oxidative stress markers and homocysteine, as well as micronutrient and carotenoid bioavailability [10,14,15,16,17,18,19,20,21]. However, this evidence was obtained mostly from younger, athlete populations and heavy smokers and over shorter time periods, while data on long-term metabolic safety (>4 months) of such supplements remain limited. Thus, existing evidence highlights the importance of long-term safety studies, particularly in older adults, as this population has been associated with more frequent use of dietary supplements [22]. In addition, older adults may be more vulnerable to adverse effects from unsafe dietary supplements, as supplement-related adverse events are common in this population [5,23]. While the health benefits of fruit and vegetable consumption are well recognized, comparable evidence on the metabolic safety of concentrated plant-based formulations consumed over long periods is lacking, particularly in older adults. Therefore, the present randomized controlled trial aimed to assess the long-term safety and tolerability of a commercially available fruit, vegetable, and berry juice powder concentrate in older adults, with metabolic safety as the primary outcome and biomarkers of systemic oxidative stress and cardiovascular health as secondary exploratory outcomes. The present work reports the pre-specified 6-month intervention timepoint of this 12-month trial, while the other intervention arms will be reported separately.

## 2. Materials and Methods

### 2.1. Study Design and Ethics

This study was designed as a randomized, controlled, open-label, parallel-group clinical trial. The full trial design has been described in detail in the published study protocol [24]. Briefly summarized, the study comprised 4 arms: control, FVB, Omega, and FVB + Omega combined. The planned sample size was 80 participants (20 per group), while the intervention was initially planned to last for 12 months, with assessments at baseline, 6 months, and 12 months. The sample size calculation was based on the primary endpoints of the overall trial, the omega-3 index and TNF-α, assuming an increase in the omega-3 index of 2.4 percentage points (SD 3.5) and a decrease in TNF-α of 0.4 ng/L (SD 0.6), with α = 0.05 and a power of 0.80, 14 participants per group were required; allowing for a drop-out rate of 30% over 12 months, 20 participants per group were planned. Accordingly, the present 6-month analysis was not powered for the parameters reported here; this is reflected in its exploratory character. All documents were approved by the Ethics Committee of the Medical University of Graz. The present analysis reports the pre-specified 6-month timepoint for the control and FVB groups, as the metabolic safety of the phytochemical-rich FVB concentrate was the focus of this evaluation; the omega-containing arms and the full two-factorial analysis will be reported separately for the complete 12-month dataset.

Participants were allocated by centralized, sex-stratified block randomization (block size 4) using Randlist software, version 1.2 (DatInf GmbH, Tübingen, Germany), performed by Green Beat Institute as the coordinating center. In the control group, participants had to maintain their habitual diet and lifestyle, whereas in the fruit-vegetable-berry group (FVB), participants were instructed to ingest a fruit, vegetable, and berry supplement while otherwise continuing their habitual diet and lifestyle. Due to the open-label design, neither participants nor investigators were blinded. The certified partner laboratory performing the analysis of metabolic safety markers and homocysteine received coded samples only and was blinded to group allocation, whereas in-house analyses were not formally blinded.

All study visits took place at the Otto Loewi Research Center, Medical University of Graz. Adverse events were recorded non-systematically by spontaneous report to the study physician, who was available throughout the trial, and were reviewed at each visit in accordance with Good Clinical Practice guidelines. This study was conducted in accordance with the Declaration of Helsinki. The study protocol and all study-related documents were approved by the Ethics Committee of the Medical University of Graz, Austria (approval number: 33-055 ex 20/21). All participants provided written informed consent before entering the study. The trial was prospectively registered at ClinicalTrials.gov (NCT04763291; https://clinicaltrials.gov/study/NCT04763291) on 21 February 2021, before enrollment of the first participant.

### 2.2. Participants

Participants were recruited via the network of the Green Beat Institute of Nutrient Research, the Medical University of Graz, social media, flyers, local physicians, pharmacies, and word of mouth. Prior to randomization and the baseline visit, participants were invited to a screening visit, during which each participant was assessed for eligibility and screened for medical history, blood pressure, and anthropometrics. At the screening visit, participants provided written informed consent. Screening visits took place between 5th October 2021, and 24th April 2025. The first baseline visit was conducted on 14 February 2023, whereas the last 6-month visit was completed on 13th November 2025. Recruitment continued after the first baseline visits had started.

Eligible participants were women and men aged 55–80 years, with women required to be postmenopausal, who were non-smokers and had a Body Mass Index (BMI) between 18.5 and 40 kg/m^2^. Additional inclusion criteria comprised a habitual fruit and vegetable intake ≤ 4 servings/day, medication taken for longer than three months with no alterations on the dosage, and adherence to a 6-week wash-out period for other dietary supplements not ingested for specific medical conditions. Temporary intake of some excluded drugs due to short-term illness did not necessarily result in exclusion from the study due to long intervals of blood sampling (6 months).

Exclusion criteria included age < 55 and >80; smokers or ex-smokers who quit smoking less than 3 years ago; pre- or perimenopausal women; unwillingness to stop multivitamins, multiminerals, or omega-fatty-acid supplements; histamine intolerance; and hypertension starting at grade 2 according to the classification of the European Society of Hypertension—systolic blood pressure > 160 mmHg, diastolic blood pressure > 100 mmHg. Further exclusion criteria included all medication taken for less than 3 months or with changes on the dosage over the last 3 months and medication for any of the conditions as follows—clinically relevant infectious diseases, all forms of dementia and Alzheimer’s disease, diabetes mellitus type I and type II, rheumatic diseases, auto-immune diseases, any stents or cardiovascular diseases, cancer patients, pregnancy, significant changes in lifestyle, diet, or physical activity profile. Accordingly, participants were considered generally healthy if none of the conditions listed above were present. Stable long-term medication not prescribed for any of the excluded conditions was permitted, whereas anticoagulant and anti-inflammatory drugs, as well as vitamin, mineral, and omega fatty acid supplements, were prohibited.

### 2.3. Study Procedures

At the screening visit, participants were informed about all study procedures, had the opportunity to ask questions to the study physician or the researchers and provided written informed consent prior to the following assessments of medical history, blood pressure, height, weight and BMI. Blood pressure was measured in a seated position after a standardized rest of 10 to 15 min using an automated oscillometric upper-arm device (boso medicus X, BOSCH + SOHN GmbH u. Co. KG, Jungingen, Germany) with the standard cuff CA 01. Measurements were performed twice on the left arm and twice on the right arm, while the mean of all measurements was used for analysis. At the baseline visit, participants were interviewed by the study physician to reconfirm eligibility and medical status. Anthropometrics were measured before fasting venous blood samples were drawn in a supine position for later analysis of blood biomarkers of metabolic safety, cardiovascular health and plasma vitamin C as a compliance marker. The participants also had to submit a detailed 4-day food diary, which they had followed in the last 4 days before the visit. At the 6-month follow-up visit, all procedures were identical, and participants had to replicate their 4-day food diary from the baseline visit to reduce nutritional bias.

### 2.4. Intervention

Participants in the FVB group were instructed to ingest 6 capsules of an encapsulated fruit, vegetable, and berry juice concentrate (Juice Plus+^®^ Premium, The Juice Plus+ Company, Memphis, TN, USA), corresponding to approximately 4.9 g of juice powder per day. One capsule of each blend was ingested with the first meal of the day and one of each blend with the second meal of the day, resulting in a total of 6 capsules per day (2 × fruit, 2 × vegetable, 2 × berry).

The fruit, vegetable, and berry blend comprises 36 fruits, vegetables, and berries, of which 6 capsules provide 1.46 mg vitamin A (retinol activity equivalents), 158 mg vitamin C, 18.65 mg α-tocopherol equivalents (α-TE, vitamin E), 316 μg folic acid, 6.1 mg lutein, and 1 mg lycopene. The (poly)phenolic composition of the concentrate has been characterized previously, and 119 different (poly)phenolic compounds were identified among the three blends of the product by mass spectrometry. Among these compounds, ellagitannins, gallotannins, dihydrochalcones, flavan-3-ols (including proanthocyanidins), flavonols, flavanones, flavones, anthocyanins, hydroxybenzoic acids, hydroxycinnamic acids, phenylethanoids, and lignans were characterized. The daily dose of 6 capsules corresponds to approximately 600 mg of (poly)phenolic compounds [25].

Compliance was assessed by (a) capsule count of returned supplement containers at each study visit and (b) verified by plasma vitamin C concentrations.

Participants allocated to the control group were instructed to maintain their habitual diet and lifestyle, without taking any dietary supplements. Study visits and blood sampling procedures were identical to those performed in the FVB group.

### 2.5. Outcomes and Measurements

For the present 6-month analysis focusing on the metabolic safety of the FVB concentrate, metabolic safety and overall tolerability were considered the primary outcome, while cardiovascular health markers were set as secondary exploratory outcomes.

Metabolic Safety: Standard clinical markers of hepatic function included aspartate aminotransferase (AST/GOT), γ-glutamyl transferase (γGT), and alkaline phosphatase (ALP). Urea, creatinine, and uric acid were used as markers of renal function. Thyroid health was assessed by measuring thyroid-stimulating hormone (TSH). Blood lipid analysis included total cholesterol, HDL, LDL, and triglycerides, whereas fasting glucose and HbA1c were assessed as biomarkers of glucose metabolism.

Markers of systemic oxidative stress and cardiovascular health comprised carbonyl proteins (CP), free malondialdehyde (MDA), oxidized LDL (oxLDL), and systolic and diastolic blood pressure, as well as homocysteine.

Plasma concentrations of vitamin C were measured as a marker of compliance and bioavailability.

### 2.6. Blood Sampling

Fasting venous blood samples were drawn by venipuncture at baseline and the 6-month follow-up visit. All blood samples were processed directly after collection. All samples that were analyzed in-house were centrifuged at 1100× *g* for 10 min to obtain the plasma before they were aliquoted accordingly and frozen at −80 °C. Samples for the measurement of safety parameters, lipid profile, fasting glucose, and homocysteine were directly transported to a certified partner laboratory. EDTA plasma was used for the analysis of biomarkers of cardiovascular health, including CP, MDA, and oxLDL.

### 2.7. Laboratory Analysis

Standard clinical safety parameters, including markers of hepatic, renal, and thyroid function, as well as glucose metabolism and lipid profile, were analyzed using routine automated clinical chemistry platforms in a certified collaborating laboratory (Lorenz & Petek, Medical and Chemical Laboratory Diagnostics, Graz, Austria) and in accordance with the manufacturers’ instructions.

CP were analyzed in EDTA plasma by an enzyme-linked immunosorbent assay (ELISA)-based detection of 2,4-dinitrophenylhydrazine (DNPH)-derivatized protein carbonyls (Carbonyl Protein ELISA Kit, Immundiagnostik AG, Bensheim, Germany). Free MDA was quantified in plasma by high-performance liquid chromatography (HPLC) following derivatization with DNPH, including external calibration with MDA standards and chromatographic separation using a reversed-phase column (ODS Hypersil, Thermo Electron Corporation, Cheshire, UK) on an HPLC system from VWR Hitachi (Vienna, Austria). oxLDL concentrations were measured in EDTA plasma using a validated solid-phase two-site ELISA (Mercodia, Uppsala, Sweden). Plasma homocysteine concentrations were determined as part of routine clinical chemistry analysis in a certified laboratory (Lorenz & Petek, Medical and Chemical Laboratory Diagnostics, Graz, Austria). Plasma vitamin C concentrations were assessed using reverse-phase HPLC at the same laboratory.

### 2.8. Statistical Analysis

Statistical analyses were performed using SPSS for Windows, version 30.0 (IBM Corp., Armonk, NY, USA). Data were tested for normality using the Shapiro–Wilk test, while homogeneity of variances was assessed by the Levene test; in case of violation, Welch’s correction was applied. For datasets violating the assumption of normality, logarithmic transformation was applied. If normality was still not achieved after transformation, non-parametric tests were used. Statistical analyses were conducted on transformed values, while original values were used for descriptive statistics.

Baseline comparisons between groups were conducted using the independent samples *t*-test or Mann–Whitney U test for non-normally distributed variables. Intervention effects were primarily evaluated using repeated measures analysis of variance (ANOVA), with time (baseline vs. 6 months) as the within-subject factor and group (control vs. FVB) as the between-subject factor. If interaction effects were significant, Bonferroni post hoc tests were applied. In addition, between-group differences in the change from baseline to 6 months were analyzed by independent samples *t*-test and are reported as mean differences with 95% confidence intervals. Furthermore, 6-month values of the cardiovascular-related markers were analyzed by analysis of covariance (ANCOVA) with the respective baseline value, age and BMI as covariates.

Within-group changes from baseline to 6 months were additionally assessed using paired samples *t*-tests or Wilcoxon signed-rank tests for variables violating the assumption of normality. Bonferroni correction was applied for multiple comparisons within each parameter, whereas no global adjustment was applied across the full panel of parameters; safety endpoints were analyzed unadjusted in order not to overlook potential adverse signals, and cardiovascular markers were regarded as exploratory. Statistical significance was set at *p* < 0.05. Analyses included all participants who completed both the baseline and the 6-month visit, and no imputation of missing data was performed; the number of participants analyzed varied between parameters due to missing individual laboratory values. Correlation analyses between outcome parameters were pre-specified in the study protocol [24] and were performed on change scores (6 months minus baseline) using Pearson correlation coefficients, pooled across both study groups.

## 3. Results

### 3.1. Baseline Characteristics

**Figure 1:**
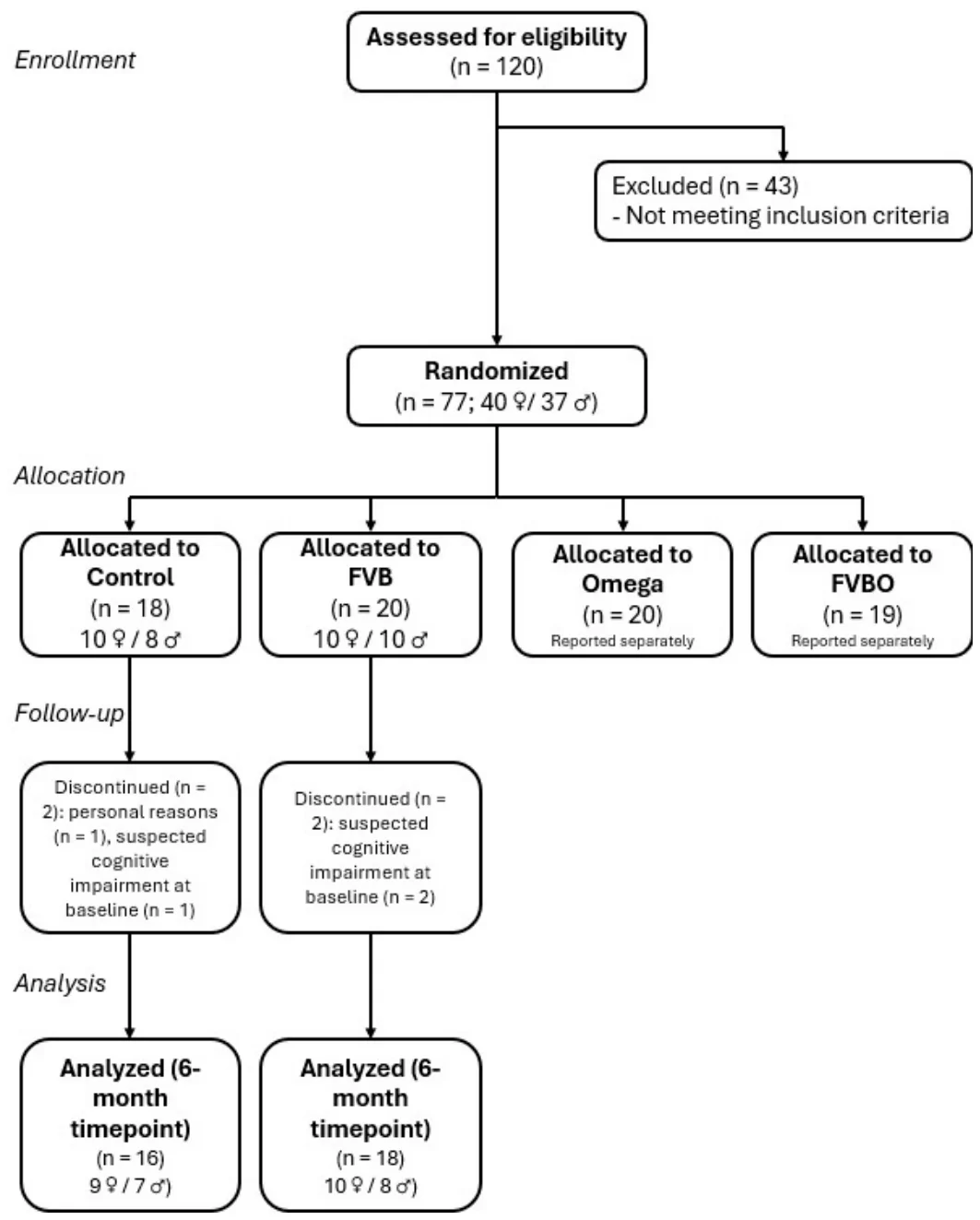
CONSORT flow diagram of participant screening, randomization, and analysis at the 6-month timepoint. ♀, female; ♂, male.

In total, 34 participants were included in the present analysis, with 16 participants allocated to the control group and 18 participants to the FVB group. The flow of participants through the study is visualized in Figure 1.

The baseline characteristics of the study population are summarized in Table 1. Of all parameters included in the analysis, no significant differences were found between groups at baseline. Fruit and vegetable intake of participants, as assessed by food diaries, averaged less than two servings per day in both the control and the FVB group.

### 3.2. Compliance and Tolerability

The FVB supplement was well tolerated, as no severe or product-related adverse events were reported during the 6-month intervention. Compliance determined by capsule count and vitamin C concentration in plasma suggested overall adherence of approximately 90%. Plasma vitamin C levels showed a significant time × group interaction (*p* = 0.004; see Table 2). Within-group analysis showed a significant increase in plasma vitamin C concentrations between baseline and 6 months in the FVB group (1.07 ± 0.28 vs. 1.38 ± 0.47 mg/dL; *p* = 0.002), while no changes were observed in the control.

In the adjusted analysis, the between-group difference in plasma vitamin C showed a trend towards significance (adjusted means 1.12 vs. 1.40 mg/dL; *p* = 0.053), while the within-group increase in the FVB group and the capsule count consistently indicated good adherence.

### 3.3. Metabolic Safety

#### 3.3.1. Hepatic Function

Biomarkers of hepatic function were assessed at baseline and after 6 months in both groups. For AST/GOT, a significant effect of time was found (*p* = 0.008). Pairwise comparisons revealed a significant decrease from baseline to the 6-month follow-up in the FVB group (28.50 ± 7.35 vs. 24.94 ± 5.88 U/L; *p* = 0.008).

For γGT, a trend towards a significant time × group interaction was detected (*p* = 0.055), which was accompanied by a trend towards a within-group decrease in the FVB group between baseline and 6 months (29.89 ± 14.37 vs. 27.50 ± 12.46 U/L; *p* = 0.172).

ALP values showed a significant time × group interaction (*p* = 0.041) and a significant increase between baseline and 6 months in the control group (72.06 ± 17.74 vs. 76.81 ± 16.86 U/L; *p* = 0.036).

Although significant effects were observed, concentrations of all markers assessed remained within the reference range.

#### 3.3.2. Renal Function

Parameters of renal function were assessed at baseline and after 6 months of intervention in both study groups. Urea showed a significant effect of time (*p* = 0.019), and within-group analysis yielded a significant increase after 6 months in the control group (27.38 ± 4.44 vs. 30.44 ± 4.66 mg/dL; *p* = 0.044). No significant changes were observed for uric acid and creatinine in either repeated measures ANOVA or within-group analysis. Despite the significant increase in urea observed in participants in the control group, values remained within the physiological reference range at both timepoints. The main results are summarized in Table 2.

#### 3.3.3. Thyroid Function and Glucose Metabolism

No significant changes were observed for TSH, as a parameter of thyroid function, nor for fasting glucose and HbA1c. Means and interaction *p*-values are shown in Table 2.

#### 3.3.4. Blood Lipids

Blood lipids, including total cholesterol, HDL, LDL, and triglycerides, were also assessed at baseline and after 6 months. No significant effects of time or time × group interactions were observed for any of these parameters.

Notably, among all safety markers assessed, only ALP and urea showed increases in within-group analysis in the control, whereas FVB group levels remained stable or decreased. However, all values remained within physiological reference ranges. A summary of all markers is visualized in Table 2.

### 3.4. Markers of Systemic Oxidative Stress and Cardiovascular Health

For CP, a significant effect of time (*p* = 0.008) and a significant time × group interaction (*p* = 0.030) were observed. Within-group analysis revealed a significant reduction in CP concentrations in the FVB group (87.60 ± 27.95 vs. 69.98 ± 16.22 U/mL; *p* = 0.020) between baseline and 6 months.

For MDA and oxLDL, no significant effects were observed in repeated-measures ANOVA and within-group analyses.

Systolic blood pressure showed a significant time × group interaction (*p* = 0.036). Within-group analysis revealed a significant reduction in the intervention group (144.24 ± 17.90 mmHg vs. 136.59 ± 10.86 mmHg; *p* = 0.018), while no changes were observed in the control group.

Diastolic blood pressure also showed a significant time × group interaction (*p* = 0.011). Pairwise comparisons indicated a non-significant decrease in the FVB group (92.15 ± 8.51 mmHg vs. 88.16 ± 6.74 mmHg; *p* = 0.058), whereas no change was observed in the control group.

For homocysteine, a significant time × group interaction was observed (*p* = 0.002). Within-group analyses revealed no significant change in the FVB group but a significant increase after 6 months in the control group (10.78 ± 2.30 vs. 11.97 ± 2.36 µmol/L; *p* = 0.006).

**Figure 2:**
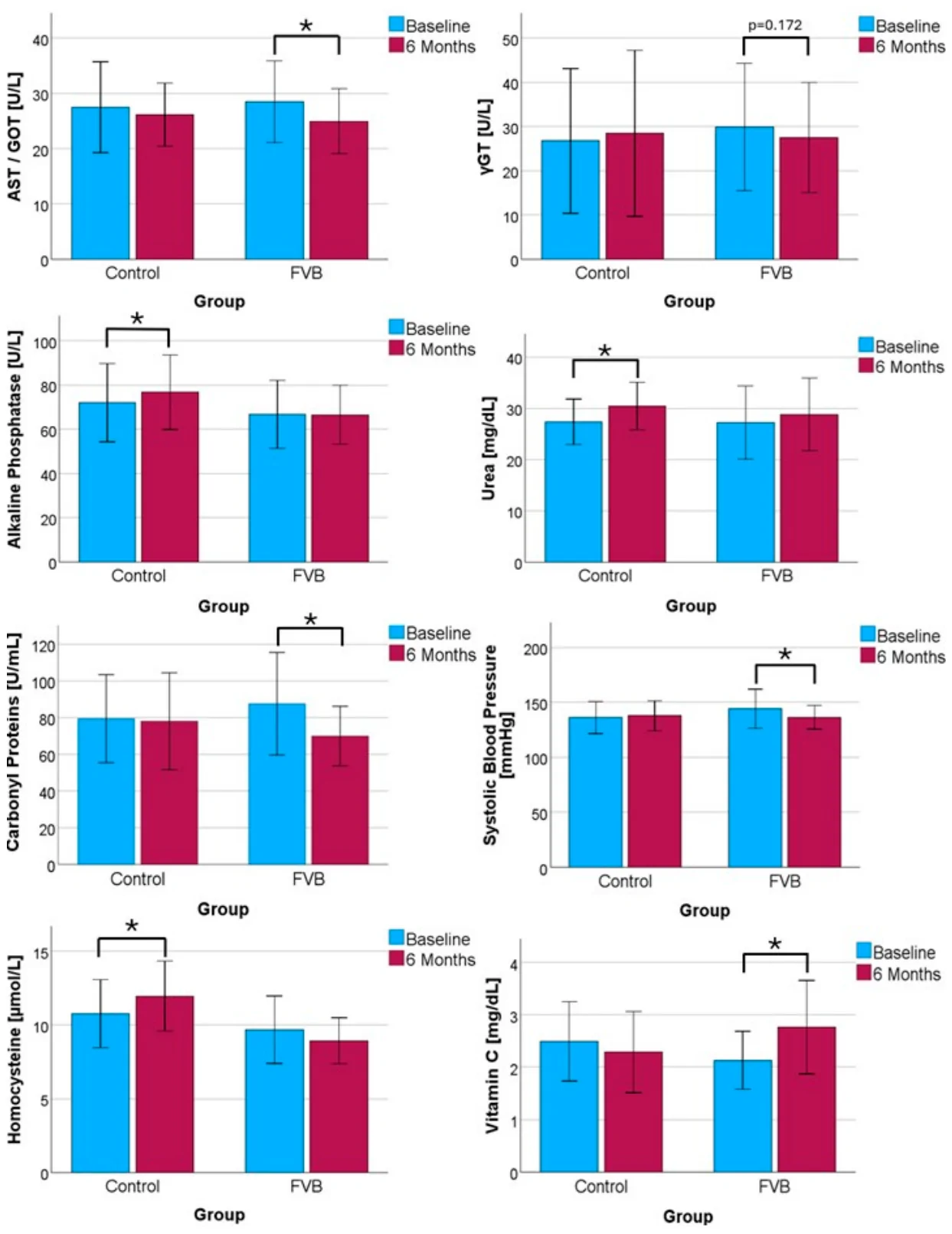
Changes in selected biomarkers of metabolic safety, cardiovascular health, and compliance following 6 months of supplementation with a fruit, vegetable, and berry juice powder concentrate. Data are presented as mean ± SD for the control and the FVB group at baseline and after 6 months. Panels display AST/GOT, γGT, ALP, urea, CP, systolic blood pressure, homocysteine, and vitamin C concentrations. Asterisks indicate significant within-group changes (paired *t*-test, * *p* < 0.05). Between-group intervention effects were tested by repeated measures ANOVA; significant time × group interactions are reported in the main text.

After adjustment for the respective baseline value, age and BMI, the between-group difference remained significant for homocysteine (adjusted means 11.57 vs. 9.29 µmol/L; *p* < 0.001, partial η^2^ = 0.394) and diastolic blood pressure (92.64 vs. 87.72 mmHg; *p* = 0.015, partial η^2^ = 0.192). For carbonyl proteins, the difference was no longer significant but still showed a trend to decrease (80.30 vs. 67.99 U/mL; *p* = 0.097, partial η^2^ = 0.099). For systolic blood pressure, adjustment for baseline values did not reveal a statistically significant between-group difference (140.03 vs. 134.48 mmHg; *p* = 0.139). Age and BMI were not significant covariates in any of the models (all *p* > 0.14). Key findings are displayed in Figure 2.

### 3.5. Correlation Analysis

Correlation analysis of change scores revealed a significant inverse association between changes in plasma vitamin C and homocysteine (r = −0.364, *p* = 0.041), which is in line with the opposing directions of these two markers observed in the FVB group.

## 4. Discussion

The present randomized controlled trial in older adults with a low habitual fruit and vegetable intake (less than two servings per day) suggests that ingestion of the investigated fruit, vegetable, and berry juice powder concentrate over a period of 6 months was well tolerated with respect to the assessed biomarkers. Although statistically significant changes were observed in safety biomarkers, none of these alterations were considered clinically relevant, as all values remained within the physiological reference range of the respective parameters. The observed reductions in AST/GOT, accompanied by a trend toward lower γGT, indicate a favorable modulation of hepatic biomarkers, probably related to the antioxidant potential of the FVB product. The increases in ALP and urea observed in the control group are consistent with age-related drifts reported for these parameters in older populations. Increases in ALP have been described, particularly in postmenopausal women, due to increased bone turnover, while urea concentrations are known to be a more sensitive indicator of subclinical age-related changes in renal function than creatinine in older adults [26,27]. Furthermore, short-term nutritional impact, e.g., increased or exaggerated protein consumption, can be excluded, as participants’ diet was monitored during the 4 days before each blood sampling without observed differences between baseline and the 6-month check. Overall tolerability is further supported by the absence of any reported severe adverse events. The findings of the present study are consistent with those of previous studies, in which no clinically relevant changes in parameters of hepatic or renal function, blood lipids, fasting glucose, HbA1c and TSH were observed, indicating good overall tolerability of the product with respect to assessed markers. Notably, these earlier investigations evaluated supplementation periods ranging from 8 to 28 weeks and were conducted in cohorts of generally healthy male and female adults, as well as trained special forces personnel aged 18 to 65 years [9,10,14,15]. In contrast, the present study extends these observations to a longer intervention period of 6 months in an older population.

The reduction of approximately 20% in CP concentrations observed in the present study is consistent with findings of prior investigations, in which intervention with the fruit, vegetable, and berry supplement generally reduced CP or attenuated their exercise-induced increase. However, these previous studies utilized exercise as a stressor to induce protein oxidation and were conducted in younger populations, whereas the present study did not apply any external stressor [9,28,29,30,31]. The between-group differences in CP were no longer statistically significant after adjustment for baseline values, age, and BMI (*p* = 0.097); however, a trend still remained, so we suggest not ignoring this observation. The reduction in CP concentrations may be related to the antioxidant and polyphenol content of the fruit, vegetable, and berry supplement, as previously characterized [25,32]. For other markers of systemic oxidative stress, MDA and oxLDL, no changes were observed, although decreases in these markers have been reported in preceding studies. For instance, in heavy smokers and obese women, supplementation with fruit, vegetable, and berry concentrates resulted in reductions in oxLDL and free MDA. In contrast to our study, these studies investigated smokers and obese individuals, characterized by elevated baseline oxidative stress, or subjects were exposed to an additional exercise stimulus like treadmill walking [30,33].

Both systolic and diastolic blood pressure decreased in the FVB group (−7.7 mmHg and −4.0 mmHg, respectively), from a baseline mean corresponding to grade 1 hypertension according to the European Society of Hypertension classification, which is consistent with previous findings investigating the same product [34]. After adjustment for baseline values, age, and BMI, the between-group difference persisted only for diastolic blood pressure (*p* = 0.015), whereas it was no longer significant for systolic blood pressure (*p* = 0.139). The reduction in diastolic blood pressure may be attributed to the flavan-3-ol and anthocyanin content of the berry and fruit blends [25], which have been associated with improved endothelial function and nitric oxide-mediated vasodilation [35].

Interestingly, homocysteine concentrations increased significantly over time in the control group, whereas levels remained stable in the FVB group. This observed pattern differs from previous studies, in which supplementation with fruit, vegetable, and berry concentrates was associated with reductions in homocysteine. Notably, most studies assessing homocysteine have been conducted in generally younger and/or fitter and/or high-risk populations [19,20,21]. The results therefore suggest that the supplement may contribute to the stabilization of homocysteine levels and potentially attenuate age-related increases, especially when intake of antioxidant- and folate-rich fruit and vegetables is low, as in the investigated cohort. This is also supported by the inverse association between vitamin C and homocysteine (r = −0.364, *p* = 0.041), suggesting the increased antioxidant capacity may contribute to the stabilization of homocysteine concentrations. Mechanistically, this stabilization could also be related to the folate content of the product [36]. This observation was robust to adjustment for baseline values, age, and BMI (*p* < 0.001).

For determination of overall compliance, we decided to use plasma vitamin C concentrations as the most appropriate parameter because several earlier studies reported increases in plasma vitamin C concentrations in generally healthy adults aged 18–60 years [14,15,16,20,37,38]. Less than 10% of returned capsules confirm the participants’ high adherence to the study protocol, which was further confirmed by the significant increase in plasma concentrations of vitamin C in the intervention group, which may additionally contribute to individual antioxidant capacity.

The long intervention period of 6 months and the randomized controlled trial design strengthen the findings of the present study. To our knowledge, this is the first study to investigate this specific fruit, vegetable, and berry supplement in older adults aged 55 to 80 years over an extended intervention period. Furthermore, the study provides a comprehensive safety evaluation of the supplement, including biomarkers of hepatic and renal function, thyroid health, glucose metabolism and blood lipids. Blood pressure was measured under standardized conditions with an automated oscillometric device, which reduces the potential for observer bias, despite the open-label design. Adherence was high and was verified by two independent measures: capsule count and plasma vitamin C concentrations. In contrast to previous studies, markers of systemic oxidative stress and cardiovascular health were assessed under resting conditions, thereby extending knowledge on potential effects independent of exercise-induced effects. The robustness of the main findings was additionally examined in covariate-adjusted analyses, which allowed for differentiation between observations that persisted after adjustment for baseline values, age, and BMI and those that did not.

However, several limitations should be acknowledged. These include moderate sample size and the open-label study design. As the sample size calculation of the complete 12-month trial was based on other primary endpoints, the present 6-month interim analysis was not powered for the parameters reported here. Nevertheless, the safety aspects of the product are worth being reported as soon as possible, corresponding to the 6-month interim data assessment in this study. In contrast, the assessment of markers of systemic oxidative stress and cardiovascular health should be considered as an exploratory approach. Another limitation is reflected by the heterogeneity of the investigated subjects in terms of age and BMI: neither variable was a significant covariate in adjusted analyses. Further, an open-label trial is always confronted with the blinding issue: the analyses of metabolic safety parameters and homocysteine were blinded to group allocation, whereas the in-house measurements of oxidative stress markers were not blinded, which cannot exclude a potential source of bias. Moreover, a multi-ingredient supplement was investigated, so none of the observed effects can be linked directly to a single compound. Last but not least, the study was conducted by the institute that received funding from the study sponsor.

## 5. Conclusions

The 6-month intervention with a fruit, vegetable, and berry concentrate was well tolerated in older adults 55 to 80 years of age. No clinically relevant adverse effects were reported for biomarkers of hepatic function, renal function, thyroid health, glucose metabolism and blood lipids. Exploratory findings suggest modulation of selected biomarkers of systemic oxidative stress and cardiovascular health. The observations of the present study suggest that the investigated product is metabolically safe and well tolerated over a 6-month period in older adults, with promising effects on markers of systemic oxidative stress and cardiovascular health. Nevertheless, these findings require independent confirmation by the completed 12-month trial.

## Figures and Tables

**Table 1:** Baseline characteristics of participants. Data are represented as mean ± SD, except for sex counts. *p*-values refer to between-group comparisons at baseline (independent samples *t*-test; chi-square test for sex).

Characteristic	Control (*n* = 16)	FVB (*n* = 18)	*p*-Value
Age (years)	63.31 ± 4.66	60.72 ± 2.67	0.063
Sex (f/m, n)	9/7	10/8	0.968
BMI (kg/m^2^)	26.93 ± 4.56	25.99 ± 4.19	0.536

**Table 2:** Biomarkers of metabolic safety and cardiovascular health at baseline and after 6 months in the control and FVB groups.

Parameter	Reference Range	Time × Group *p*	Control Baseline	Control 6 Months	*p*	FVB Baseline	FVB 6 Months	*p*	Δ Difference FVB − Control (95% CI)
AST/GOT (U/L)	10–50	0.125	27.50 ± 8.22	26.13 ± 5.70	0.429	28.50 ± 7.35	24.94 ± 5.88	0.008 *	−2.18 (−5.83 to 1.47)
γGT (U/L)	<68	0.055	26.75 ± 16.34	28.44 ± 18.75	0.293	29.89 ± 14.37	27.50 ± 12.46	0.172	−4.08 (−8.72 to 0.57)
ALP (U/L)	40–150	0.041 *	72.06 ± 17.74	76.81 ± 16.86	0.036 *	66.83 ± 15.37	66.56 ± 13.28	0.862	−5.03 (−9.84 to −0.21)
Urea (mg/dL)	19–44	0.448	27.38 ± 4.44	30.44 ± 4.66	0.044 *	27.22 ± 7.15	28.83 ± 7.11	0.274	−1.45 (−5.30 to 2.40)
Uric acid (mg/dL)	3.7–7.7	0.485	5.13 ± 1.30	5.19 ± 1.44	0.664	5.53 ± 1.08	5.44 ± 0.97	0.582	−0.16 (−0.61 to 0.30)
Creatinine (mg/dL)	0.72–1.25	0.426	0.84 ± 0.12	0.84 ± 0.14	0.945	0.86 ± 0.11	0.88 ± 0.15	0.329	0.02 (−0.04 to 0.08)
TSH (mU/L)	0.25–4.04	0.770	1.85 ± 1.34	1.63 ± 0.76	0.898	1.58 ± 0.45	1.75 ± 0.85	0.756	0.38 (−0.38 to 1.15)
Fasting glucose (mg/dL)	<100	0.792	94.25 ± 7.85	92.38 ± 8.07	0.393	93.22 ± 10.47	92.00 ± 9.55	0.370	0.65 (−4.34 to 5.65)
HbA1c (%)	4–6	0.759	5.39 ± 0.23	5.41 ± 0.26	0.549	5.33 ± 0.27	5.34 ± 0.25	0.854	−0.01 (−0.10 to 0.07)
Total cholesterol (mg/dL)	<200	0.833	215.50 ± 27.66	216.25 ± 29.40	0.905	223.17 ± 36.06	222.11 ± 40.38	0.858	−1.81 (−19.15 to 15.54)
HDL (mg/dL)	>40	0.277	58.25 ± 13.07	59.56 ± 15.65	0.523	59.89 ± 15.08	58.00 ± 16.51	0.370	−3.20 (−7.86 to 1.46)
LDL (mg/dL)	<160	0.847	137.19 ± 26.31	136.75 ± 28.95	0.939	140.06 ± 31.68	141.06 ± 33.84	0.839	1.44 (−13.63 to 16.50)
Triglycerides (mg/dL)	<150	0.953	100.06 ± 33.57	99.69 ± 43.13	0.965	116.11 ± 53.44	114.94 ± 56.35	0.910	−0.79 (−28.17 to 26.59)
Carbonyl proteins (U/mL)	n.a.	0.030 *	79.44 ± 24.00	78.04 ± 26.39	0.452	87.60 ± 27.95	69.98 ± 16.22	0.020 *	−16.21 (−32.18 to −0.24) ‡
MDA (nmol/mL)	n.a.	0.862	3.40 ± 0.86	3.50 ± 1.35	0.980	4.02 ± 1.42	3.92 ± 1.19	0.839	−0.10 (−1.27 to 1.06)
oxLDL (mU/L)	n.a.	0.839	9.78 ± 2.40	10.06 ± 2.51	0.472	10.20 ± 2.73	10.37 ± 2.74	0.670	−0.12 (−1.26 to 1.03)
Systolic BP (mmHg)	n.a.	0.036 *	136.16 ± 14.61	137.80 ± 13.55	0.635	144.24 ± 17.90	136.59 ± 10.86	0.018 *	−9.29 (−17.93 to −0.65)
Diastolic BP (mmHg)	n.a.	0.011 *	90.41 ± 8.37	92.17 ± 6.96	0.191	92.15 ± 8.51	88.16 ± 6.74	0.058	−5.75 (−10.08 to −1.42)
Homocysteine (µmol/L)	5.5–16.2	0.002 *	10.78 ± 2.30	11.97 ± 2.36	0.006 *	9.68 ± 2.29	8.94 ± 1.55	0.127	−1.92 (−3.10 to −0.74)
Vitamin C (mg/dL)	0.5–1.8	0.004 *	1.25 ± 0.40	1.14 ± 0.39	0.354	1.07 ± 0.28	1.38 ± 0.47	0.002 *	0.42 (0.15 to 0.69)

Data are presented as mean ± standard deviation. The time × group *p*-value refers to repeated-measures ANOVA; within-group *p*-values represent changes from baseline to 6 months (paired samples *t*-test). The Δ difference represents between-group differences in the change scores from baseline to 6 months (FVB minus control) with its 95% confidence interval, obtained by independent samples *t*-test. For variables not meeting the assumption of normality, time × group analysis was performed on log-transformed data, while original values and confidence intervals are shown on the original scale for interpretability. Significant values (*p* < 0.05) are marked with an asterisk (*). ‡ Welch correction applied (Levene’s test *p* < 0.05). Reference ranges were provided by the analyzing laboratory (Lorenz & Petek, Medical and Chemical Laboratory Diagnostics, Graz, Austria). n.a., not applicable (no established reference range).

## Data Availability

The data reported in this study can be made available upon request from the corresponding author, as the data are not publicly available due to privacy restrictions.
